# Intrinsic capacity and risks of disability and mortality in octogenarians: a prospective analysis of two independent Japanese cohorts

**DOI:** 10.1016/j.tjfa.2026.100230

**Published:** 2026-11-05

**Authors:** Yusuke Osawa, Takashi Sasaki, Yukiko Abe, Yasumichi Arai

**Affiliations:** a Graduate School of Health Management, Keio University, Tokyo, Japan; b Center for Supercentenarian Medical Research, Keio University School of Medicine, Tokyo, Japan; c Sports Medicine Research Center, Keio University, Kanagawa, Japan; d Faculty of Nursing and Medical Care, Keio University School of Medicine, Kanagawa, Japan

**Keywords:** Intrinsic capacity, Disability, Mortality, Oldest old, Healthy aging

## Abstract

**Background:**

Intrinsic capacity (IC) is central to healthy aging, but its prognostic relevance among the oldest old remains unclear.

**Objectives:**

To investigate the associations of IC with disability and mortality, and to determine whether an exploratory cardio-renal measure adds prognostic information.

**Design:**

Prospective cohort study, with the two cohorts analyzed separately.

**Setting:**

Community-dwelling older adults in Japan.

**Participants:**

Adults aged 85–89 years from the Kawasaki Aging and Wellbeing Project (KAWP; *n* = 942) and the Tokyo Oldest-Old Survey on Total Health (TOOTH; *n* = 393).

**Measurements:**

Five IC domains were individually rescaled to a 0–100 scale and averaged, with higher scores indicating better capacity. An exploratory cardio-renal measure comprising N-terminal pro-B-type natriuretic peptide and cystatin C was entered alongside the IC score. The outcomes were incident disability (long-term care insurance certification in KAWP; activities of daily living dependency in TOOTH) and all-cause mortality. Cox proportional hazards and Fine-Gray competing-risk models were adjusted for age, sex, education, smoking status, comorbidity burden, and body mass index.

**Results:**

In KAWP, each 10-point increment in the IC score was associated with lower risks of care level 1 (adjusted hazard ratio [HR] 0.72, 95% CI 0.66–0.79) and mortality (HR 0.78, 0.68–0.90). In TOOTH, associations were comparable for mortality (HR 0.74, 0.60–0.91) and disability or death (HR 0.78, 0.67–0.91). The association with disability was no longer evident when death was treated as a competing event. The cardio-renal measure provided modest but consistent incremental information for mortality.

**Conclusions:**

Higher IC was associated with lower risks of disability and mortality among octogenarians across two independent Japanese cohorts. These findings suggest that IC captures shared vulnerability to disability and death and may serve as a population-level marker of vulnerability rather than as a tool for individual-level risk stratification.

## 1 Introduction

The concept of intrinsic capacity (IC), proposed by the World Health Organization (WHO), has emerged as a central framework for understanding healthy aging [1]. IC represents the composite of an individual’s physical and mental capacities across five domains: locomotion, cognition, psychological wellbeing, vitality and sensory function [1]. Accumulating longitudinal evidence from Asia, Europe, and Latin America indicates that lower IC is associated with an increased risk of disability, functional decline, hospitalization, and mortality among older adults [[2], [3], [4], [5]]. Large prospective cohort studies, including the China Health and Retirement Longitudinal Study, the I-Lan Longitudinal Aging Study, the English Longitudinal Study of Ageing and multinational cohorts such as the 10/66 Dementia Research Group and SHARE, have consistently demonstrated that lower IC and greater declines in IC are associated with subsequent dependence and mortality [[5], [6], [7], [8]]. Collectively, these findings support IC as a multidimensional indicator of vulnerability to adverse health outcomes.

Despite growing interest in IC, important knowledge gaps remain. The population aged 85 years and older—the oldest old—is expanding rapidly worldwide, particularly in Asia, and experiences the highest burden of disability, dependency, and mortality [2,5,9,10]. Identifying early markers of adverse health outcomes in this population is therefore critical for both public health and clinical care. However, relatively few studies have specifically examined the prognostic relevance of IC among the oldest old, who are frequently under-represented in cohorts of adults aged 60 or 65 years and older [2,5,[8], [9], [10]]. Consequently, whether the IC framework retains its prognostic relevance at very advanced ages remains incompletely understood.

Furthermore, the relative contributions of individual IC domains to adverse health outcomes remain insufficiently characterized, particularly among the oldest old. Several studies have examined associations between individual IC domains and adverse outcomes in older adults. Impairments in locomotor, cognitive, vitality and psychological domains have been associated with functional decline, disability, and mortality in community-dwelling older adults [8,9,[11], [12], [13]]. Locomotor and cognitive domains have frequently demonstrated the strongest associations with disability and survival, while vitality-related indicators, including nutritional status, have also been linked to mortality and functional deterioration [9,[13], [14], [15]]. Nevertheless, findings have been heterogeneous, and the relative importance of individual domains appears to vary according to the population and outcome examined. Moreover, most available evidence is derived from cohorts of adults aged 60 or 65 years and older. Thus, the prognostic relevance of specific IC domains may change with advancing age. Some domains may remain strongly associated with adverse outcomes, whereas others may become less informative in the oldest old. Determining which domains retain prognostic relevance could help refine the applicability of the IC framework.

In the oldest old, pervasive competing mortality and multimorbidity may also alter how IC relates to adverse outcomes. In particular, individuals with low IC may die before disability can develop or be observed. Consequently, associations between IC and non-fatal outcomes such as disability depend on how the competing risk of death is handled analytically—an issue that becomes central at these ages but is seldom examined.

A further unresolved question is whether the five WHO domains adequately capture physiological reserve in this age group. Cardiovascular and kidney dysfunction are highly prevalent among the oldest old and represent major determinants of adverse health outcomes in advanced age. In Japan, cardiovascular disease and chronic kidney disease contribute substantially to disability and mortality among adults aged 85 years and older [16,17]. Biomarkers reflecting cardio-renal physiological reserve, including N-terminal pro-B-type natriuretic peptide (NT-proBNP) and cystatin C, have been associated with frailty, functional decline, and mortality in older adults, including centenarians [[18], [19], [20]]. In our previous work in the Kawasaki Aging and Wellbeing Project (KAWP) cohort, one of the two cohorts analyzed here, higher concentrations of cystatin C and NT-proBNP were independently associated with an increased risk of incident disability. These associations were subsequently replicated in an independent Italian cohort among participants aged 80 years and older, providing a rationale for examining these two biomarkers in the present study [21]. In parallel, increasing efforts have sought to extend the WHO IC framework by broadening the vitality domain beyond nutritional and muscular measures to encompass metabolic, immune, and other biomarkers of physiological and homeostatic reserve. However, the incremental prognostic value of such extensions has generally been modest and inconsistent [22,23].

Testing such an extension, however, requires care in how the additional measure is evaluated. Adding a sixth component to the composite is the intuitive approach, but averaging over six components necessarily dilutes the weight of each of the five original domains, so any change in performance reflects both the new information and a change in the scaling of the existing domains. Entering the measure as a separate term alongside the intact five-domain score separates these two effects and asks the narrower question of whether the biomarkers carry prognostic information the five domains do not.

Accordingly, the primary objective of this study was to examine the associations of IC with disability and mortality among community-dwelling octogenarians across two independent Japanese cohorts, thereby assessing the consistency of these associations in advanced age, and to determine how these associations change when death is treated as a competing event rather than as censoring. As a secondary, exploratory objective, we examined whether a cardio-renal measure adds prognostic information beyond the five domains, evaluating it both within a recomputed six-domain composite and as a separate term entered alongside the intact five-domain IC score.

## 2 Methods

### 2.1 Study design and participants

This study used data from two prospective cohort studies of community-dwelling adults aged 85–89 years in Japan: the KAWP and the Tokyo Oldest-Old Survey on Total Health (TOOTH). The two cohorts were analyzed separately throughout and were not pooled. Several key measures were not identical across the two studies—disability, self-rated vision, and the ascertainment of death, each described below—and the baseline assessment periods differed by approximately one decade. Pooling would therefore have required combining measures that are related but not directly equivalent. KAWP recruited residents aged 85–89 years in Kawasaki City between 2017 and 2018 [24]. TOOTH is a prospective cohort study of community-dwelling adults aged ≥85 years residing in central Tokyo (Shinjuku-ku, Minato-ku, and Shibuya-ku), with baseline assessments conducted between 2008 and 2009 [25]. In both cohorts, baseline assessment comprised physical performance, cognitive, psychological and sensory measures, blood sampling, and self-reported sociodemographic and lifestyle information; the individual measures are described below. Participants were followed prospectively for disability and death; the sources used in each cohort are described below. We selected the KAWP and TOOTH studies because, to our knowledge, they are the only Japanese cohorts with harmonizable measures covering all five WHO IC domains in older adults aged 85–89 years and with prospective follow-up for disability and death. Of 542 TOOTH baseline participants, we restricted the analysis to those aged 85–89 years at baseline; 52 participants outside this range were excluded to achieve an age distribution comparable with that of KAWP. Thus, both analytic cohorts were restricted to adults aged 85–89 years. Participants were required to have complete data for all five IC domains, prespecified covariates, and outcomes because the composite IC score could not be computed when any constituent domain was missing. Among 1026 KAWP participants, 84 (8.2%) were excluded, leaving 942 participants for analysis. Among 490 age-eligible TOOTH participants, 97 (19.8%) were excluded, leaving 393 participants. Baseline characteristics of included and excluded participants were compared across available variables (**Table S1**). In KAWP, excluded participants were broadly comparable with those included, except for a lower proportion with high school education. In TOOTH, excluded participants were slightly older and had lower Mini-Mental State Examination (MMSE), WHO-5, grip strength, and vision scores, and were less likely to have completed high school. Both studies were conducted in accordance with the Declaration of Helsinki. The study protocol was approved by the respective institutional ethics committees, and all participants provided written informed consent. Both studies were registered in the University Hospital Medical Information Network Clinical Trials Registry (UMIN000026053 for KAWP and UMIN000001842 for TOOTH).

### 2.2 IC assessment

IC was assessed according to the WHO framework across five core domains [26]: locomotor, cognitive, psychological, vitality, and sensory capacity. Locomotor capacity was assessed using the Timed Up and Go (TUG) test, cognitive capacity using the MMSE, and vitality using handgrip strength (**Table S2**). To operationalize the psychological domain, we used the WHO-5 Well-being Index, the only psychological instrument administered identically in both cohorts; no single instrument has been established as standard for this domain within the IC framework. Although the WHO-5 primarily captures subjective wellbeing, it is a validated first-step screening instrument for depressive symptoms, including in Japanese populations [27,28]. Sensory capacity was assessed using self-reported vision and hearing. Hearing was assessed using a standardized four-level question in both cohorts, ranging from no difficulty in everyday conversation to complete hearing loss. Vision was assessed using cohort-specific self-rated measures: a five-level scale in KAWP and a four-level scale in TOOTH. Both scales ranged from good vision, with usual correction, to severe visual impairment or blindness. All IC domains were modeled as continuous scores rather than dichotomized into deficit present or absent, so no domain-specific cut-points were applied (**Table S2**). Raw measurements were standardized using the percentage of maximum possible method to generate scores ranging from 0 to 100, with higher scores indicating better IC [29,30].

### 2.3 Plasma biomarkers and the cardio-renal measure

Non-fasting blood samples were collected at baseline and stored at −80 °C until analysis. Plasma NT-proBNP levels were measured using an electro-chemiluminescence immunoassay, with an inter-assay coefficient of variation of 1.57%. Plasma cystatin C levels were measured using a colloidal gold immunoassay, with intra- and inter-assay coefficients of variation of 1.72% and 0.79%, respectively (SRL Inc., Tokyo, Japan). NT-proBNP and cystatin C measurements were available for all 942 KAWP participants and in 360 of the 393 TOOTH participants included in the primary analysis. Accordingly, analyses incorporating the cardio-renal measure in TOOTH were restricted to 360 participants. The cardio-renal measure was constructed by log-transforming NT-proBNP, standardizing the transformed NT-proBNP and cystatin C values, summing the standardized values, and reversing the direction so that higher scores indicated better cardio-renal reserve.

### 2.4 Composite IC scores

Two composite IC scores were derived. The conventional five-domain IC score was calculated as the arithmetic mean of locomotor, cognitive, psychological, vitality, and sensory domains. A six-domain IC score was additionally constructed by incorporating the cardio-renal measure as an equally weighted sixth component. Because adding a sixth component necessarily reduces the weight of each of the five original domains, the cardio-renal measure was also evaluated independently by entering it as a separate term alongside the intact five-domain IC score.

### 2.5 Disability and all-cause mortality

In KAWP, incident disability was identified using the Japanese Long-Term Care Insurance (LTCI) registry in Kawasaki City, and deaths and dates of death were obtained from municipal death registries in Kanagawa Prefecture. Disability was defined by LTCI certification at care levels 1 (*Yokaigo 1*) and 2 (*Yokaigo 2*). A composite outcome comprising care level 2 disability or death was also evaluated. The initial examination date was defined as the time of study entry, and participants were followed for up to 5.5 years. In TOOTH, outcomes comprised all-cause mortality and disability defined according to decline in activities of daily living (ADL). ADL was assessed using the Barthel Index at baseline and at 3- and 6-year follow-up visits. ADL disability was defined as dependency in one or more ADL items (Barthel Index <100) at follow-up. Because ADL was assessed only at discrete follow-up visits, the exact time of onset of ADL disability was not observed and was therefore interval-censored. Consequently, the composite outcome of ADL disability or death was prespecified as the primary disability-related outcome in TOOTH. Deaths in TOOTH were ascertained through annual telephone follow-up conducted from June 2009 to December 2015. The initial examination date was defined as the time of entry, with follow-up extending up to 6 years. Participants lost to follow-up were censored at the date of their last contact or follow-up visit. Outcome ascertainment was not harmonized between the two cohorts; each cohort was analyzed using its own outcome definitions.

#### 2.5.1 Covariates

Covariates were specified a priori and comprised age, sex, educational attainment, smoking status, comorbidity burden, and body mass index (BMI). Educational attainment was obtained by self-report and dichotomized as high school graduation or above versus below high school. Smoking status was self-reported and classified as ever versus never. BMI was calculated from measured height and weight. Comorbidity burden was quantified using the Charlson Comorbidity Index [31], with the dementia component excluded to minimize conceptual overlap with the cognitive IC domain.

### 2.6 Statistical analysis

Baseline characteristics were summarized separately for the KAWP and TOOTH cohorts. Continuous variables were presented as means ± standard deviations (SDs) or medians with interquartile ranges, as appropriate, and categorical variables as counts and percentages. Between-group differences were assessed using *t*-tests, Wilcoxon rank-sum tests, or chi-square tests as appropriate.

The two cohorts were analyzed separately throughout, using an identical covariate set and prespecified model specifications; data were not pooled (see Study design and participants). Applying the same analytical framework to each cohort enabled assessment of the consistency of associations across populations that differ in time period, geographic setting, and outcome ascertainment.

Associations of IC with incident disability and all-cause mortality were examined using Cox proportional hazards models. Hazard ratios (HRs) and 95% confidence intervals (CIs) were expressed per 10-point increment in the IC score; accordingly, an HR below 1 indicates a lower hazard associated with better IC. Because the composite score ranged from 0 to 100, a 10-point increment represented one-tenth of the theoretical scale and provided interpretable and directly comparable effect estimates across cohorts and individual IC domains. Models were estimated in two stages: the base model was adjusted for age and sex; and fully adjusted models were additionally adjusted for educational attainment, smoking status, comorbidity burden, and BMI. The same covariate set was applied across both cohorts. BMI was included as a confounder rather than as an IC indicator. Whereas each IC domain is scaled so that greater values indicate better capacity, the association between BMI and health is non-monotonic (U-shaped, with both low and high BMI conferring risk), and thus, we used grip strength to operationalize the vitality domain and utilized BMI as a covariate.

The incremental prognostic contribution of the cardio-renal measure was examined in two ways. First, an extended six-domain composite was constructed by adding the cardio-renal measure as an equally weighted sixth component, and its discrimination was compared with that of the conventional five-domain IC scores. Second, because incorporating an additional component changes the relative weighting of the five original domains, nested models were fitted in which the intact five-domain IC score and the standardized cardio-renal measure (per 1 SD) were entered as separate terms. Model discrimination was assessed using Harrell’s concordance index (C-index) and model prediction error was assessed using the integrated Brier score [32,33]. The scaled Brier score, calculated as 1 – (model Brier score/null-model Brier score), was used as a measure of predictive accuracy [34,35]. Differences in C-index (ΔC-index) between models were accompanied by bootstrap 95% CIs based on 1000 resamples. To account for the competing risk of death when modeling disability, Fine-Gray subdistribution hazard models were fitted with death treated as the competing event [36]. Kaplan–Meier curves were generated to visualize the cumulative incidence of disability and all-cause mortality according to tertiles of the five-domain IC score, with differences between tertiles assessed using the log-rank test. Domain-specific associations were examined by entering each standardized domain score separately into Cox models adjusted for the same covariate set as the fully adjusted models. All statistical analyses were conducted using R version 4.4.2 (R Foundation for Statistical Computing, Vienna, Austria). The analysis plan and all statistical decisions, including model specification, covariate selection and the handling of competing risks, were determined by the authors before analysis. Generative AI (Claude Opus 5, Anthropic) was used solely to assist with developing and debugging the R code used for the statistical analyses (Fine-Gray competing-risk models and model calibration). The first author reviewed the code line by line against the analysis plan, and a co-author independently verified the reported estimates against the analysis output. All authors take full responsibility for the analyses. All tests were two-sided, and *P* values less than 0.05 were considered statistically significant.

## 3 Results

### 3.1 Participant characteristics

In total, 942 participants from KAWP and 393 participants from TOOTH were included (Fig. 1). Baseline characteristics of both cohorts are summarized in Table 1. KAWP participants were slightly younger than TOOTH participants (86.6 *vs*. 87.3 years) and had better physical performance (i.e., grip strength and TUG), whereas their MMSE scores were lower. TOOTH participants had higher cardio-renal-related biomarker levels. A significant difference in IC scores was found between cohorts (KAWP: 65.1 *vs*. TOOTH: 75.0; *p* < 0.001).

**Fig. 1:**
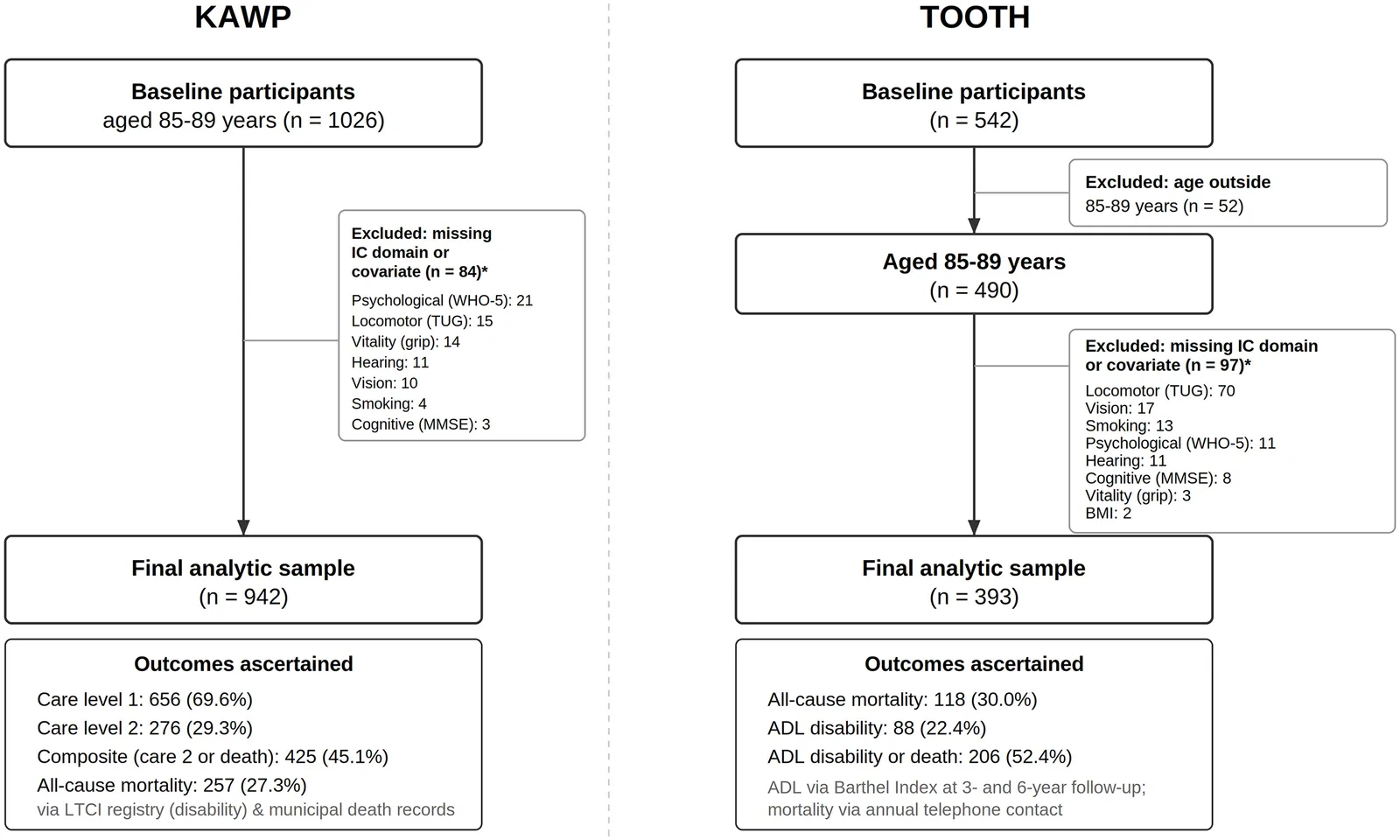
Flow chart of participant selection in the Kawasaki Aging and Wellbeing Project (A) and the Tokyo Oldest-Old Survey on Total Health (B)" *Numbers of missing values are shown for each variable and may overlap, because an individual participant could have missing data for more than one variable. Participants were excluded if any intrinsic capacity component or covariate required for the fully adjusted analysis was missing. Abbreviations: KAWP, Kawasaki Ageing and Wellbeing Project; TOOTH, Tokyo Oldest-Old Survey on Total Health; IC, intrinsic capacity; TUG, Timed Up and Go test; WHO-5, WHO-5 Well-being Index; MMSE, Mini-Mental State Examination; NT-proBNP, N-terminal pro-B-type natriuretic peptide; LTCI, Long-Term Care Insurance; ADL, activities of daily living.

**Table 1:** Participant characteristics in KAWP and TOOTH studies.

Variable	KAWP	TOOTH	p-value
N	942	393	
Age, years, mean (SD)	86.6 (1.4)	87.3 (1.3)	<0.001
Female, n (%)	480 (51.0)	218 (55.5)	0.148
BMI, kg/m^2^, mean (SD)	23.3 (3.1)	21.6 (3.2)	<0.001
Education (high school+), n (%)	382 (40.6)	151 (38.4)	0.507
Ever smoker, n (%)	394 (41.8)	162 (41.2)	0.886
CCI, median [IQR]	2.0 [1.0 − 3.0]	2.0 [1.0 − 3.0]	<0.001
IC 5-domain score (0–100), mean (SD)	65.1 (8.6)	75.0 (8.9)	<0.001
IC 6-domain score (0–100), mean (SD)	66.5 (7.6)	75.6 (7.9)	<0.001
— IC component measures (original units) —			
Locomotor: TUG, *sec*, mean (SD)	11.6 (2.9)	14.9 (8.7)	<0.001
Cognitive: MMSE, points, mean (SD)	26.1 (2.8)	26.8 (3.2)	<0.001
Psychological: WHO-5, points, mean (SD)	18.3 (5.1)	18.7 (5.2)	0.178
Vitality: Grip strength, kg, mean (SD)	23.5 (6.3)	20.6 (5.6)	<0.001
Sensory: Vision score, median [IQR]	3 [3 − 3]	4 [4 − 4]	
Sensory: Hearing score, median [IQR]	3 [3 − 4]	3 [2 − 3]	
Cardio-renal: NT-proBNP, pg/mL, median [IQR]	144.0 [87.0 − 258.8]	191.0 [113.5 − 376.5]	<0.001
Cardio-renal: Cystatin C, mg/L, median [IQR]	1.09 [0.95−1.26]	1.17 [1.02−1.38]	<0.001
— Outcomes —			
Care level 1, n (%)	656 (69.6)	—	
Care level 2, n (%)	276 (29.3)	—	
Composite (care level 2/death), n (%)	425 (45.1)	—	
All-cause mortality, n (%)	257 (27.3)	118 (30.0)	
ADL disability, n (%)	—	88 (22.4)	
ADL disability or death, n (%)	—	206 (52.4)	

Values are mean (SD), median [IQR], or n (%). P-values compare KAWP and TOOTH (Wilcoxon rank-sum or chi-square test).

IC domain and total scores were standardized within each cohort to 0–100 (percentage of maximum possible); component measures are shown in original units.

Vision and hearing scores are reverse-coded so that higher indicates better function, but the underlying scales differ between cohorts (vision: 5 levels in KAWP, 4 levels in TOOTH); they are therefore not compared between cohorts.

Care levels 1 and 2 and the composite were defined only in KAWP; ADL disability outcomes only in TOOTH. Outcome frequencies were not compared between cohorts because follow-up duration differed (KAWP, up to 5.5 years; TOOTH, up to 6 years).

Abbreviations: ADL, activities of daily living; BMI, body mass index; CCI, Charlson Comorbidity Index; IC, intrinsic capacity; IQR, interquartile range; MMSE, Mini-Mental State Examination; NT-proBNP, N-terminal pro-B-type natriuretic peptide; SD, standard deviation; TUG, Timed Up and Go test; WHO-5, WHO-5 Well-being Index.

### 3.2 Association between individual IC domains and adverse outcomes

Fig. 2 presents the domain-specific associations of IC with disability and all-cause mortality in the two cohorts. Each domain was entered into a separate model adjusted for the same covariates. These models describe the association of each domain considered on its own and do not identify its independent contribution after accounting for the other domains. In KAWP, higher scores in most IC domains were associated with lower risks of adverse outcomes. Locomotor capacity demonstrated the strongest association with incident disability (HR per 10-point higher score for care level 1, 0.79, 95% CI 0.75–0.84), followed by vitality (HR 0.91, 0.85–0.97) and cognitive capacity (HR 0.92, 0.87–0.98), whereas the associations with psychological and sensory domains were weaker and less consistent, particularly for care level 2 disability. The cardio-renal measure was significantly associated with disability and mortality in KAWP. In TOOTH, vitality (HR for all-cause mortality 0.75, 0.63–0.89), cognitive capacity (HR 0.81, 0.72–0.91), and locomotor capacity (HR 0.80, 0.71–0.90) were associated with mortality and with the composite of ADL disability or death.

**Fig. 2:**
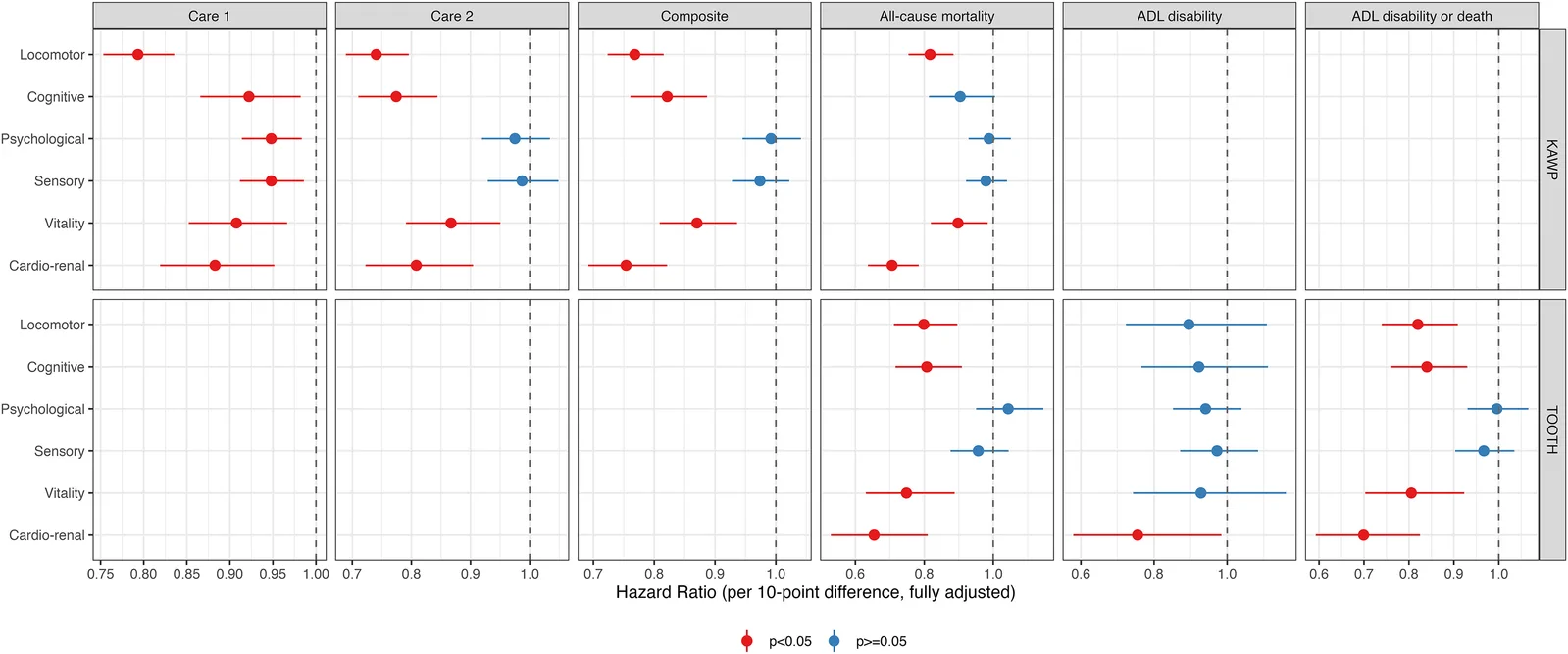
Forest plots showing the associations of individual intrinsic capacity domains with the risk of disability and all-cause mortality in the Kawasaki Ageing and Wellbeing Project and the Tokyo Oldest-Old Survey on Total Health Abbreviations: KAWP, Kawasaki Aging and Wellbeing Project; TOOTH, Tokyo Oldest-Old Survey on Total Health; ADL, activities of daily living.

### 3.3 Kaplan–Meier analysis by IC tertiles

Fig. 3 displays Kaplan–Meier curves for disability and all-cause mortality, stratified by five-domain IC tertiles in KAWP. Participants in the lowest IC tertile exhibited the highest cumulative incidence of disability and mortality, whereas those in the highest tertile had the lowest risk (all log-rank *p* < 0.05). Corresponding Kaplan–Meier curves for TOOTH are presented in Figure S1.

**Fig. 3:**
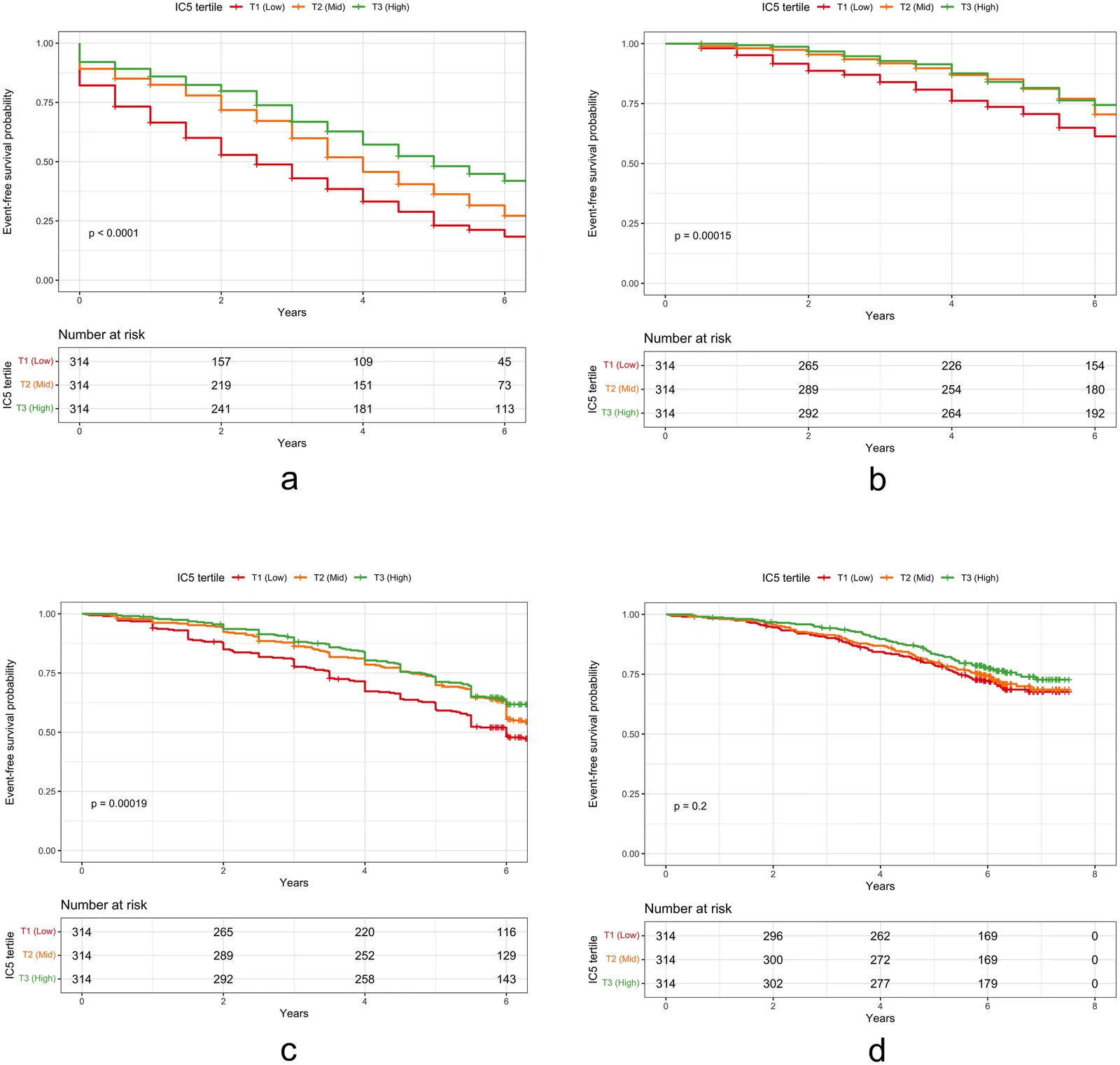
Kaplan–Meier curves of the five-domain intrinsic capacity score, categorized by tertiles, according to disability and all-cause mortality in the Kawasaki Ageing and Wellbeing Project (a) Care level 1; (b) care level 2; (c) composite outcome of care level 2 disability or death; and (d) all-cause mortality. Abbreviations: IC, intrinsic capacity.

### 3.4 Associations of the five-domain IC score with adverse outcomes

Table 2 summarizes the associations of the five-domain IC score with adverse outcomes. In KAWP, each 10-point increment in the five-domain IC score was associated with substantially lower risk of care level 1 disability (HR 0.72, 0.66–0.79), care level 2 disability (HR 0.67, 0.58–0.77), the composite outcome (HR 0.71, 0.64–0.79), and all-cause mortality (HR 0.78, 0.68–0.90). Associations changed little after adjustment for additional covariates. Discrimination was modest, with C-indices ranging from 0.61 to 0.67 in fully adjusted models in KAWP.

**Table 2:** Associations of the five-domain intrinsic capacity score with incident disability and all-cause mortality in the KAWP and TOOTH studies.

				Base model			Fully adjusted model		
Cohort	Outcome	N	Events	HR (95%CI)	p-value	C-index	HR	p-value	C-index
KAWP	Care level 1	942	656	0.72 (0.66−0.79)	<0.001	0.607	0.72 (0.66−0.79)	<0.001	0.610
	Care level 2	942	276	0.68 (0.59−0.77)	<0.001	0.606	0.67 (0.58−0.77)	<0.001	0.611
	Composite	942	425	0.70 (0.63−0.78)	<0.001	0.609	0.71 (0.64−0.79)	<0.001	0.615
	All-cause mortality	942	257	0.75 (0.66−0.87)	<0.001	0.647	0.78 (0.68−0.90)	0.001	0.666
TOOTH	All-cause mortality	393	118	0.74 (0.61−0.90)	0.003	0.604	0.74 (0.60−0.91)	0.005	0.619
	ADL disability	393	88	0.80 (0.63−1.02)	0.073	0.671	0.84 (0.66−1.07)	0.159	0.692
	ADL disability or death	393	206	0.77 (0.66−0.89)	0.001	0.580	0.78 (0.67−0.91)	0.002	0.605

Hazard ratios (HR) and 95% confidence intervals (CI) are expressed per 10-point difference in the five-domain intrinsic capacity (IC) score.

Base model: adjusted for age and sex. Fully adjusted model: additionally adjusted for education (high school or above), smoking status (ever vs never), Charlson Comorbidity Index, and body mass index. The same covariate set was applied in KAWP and TOOTH.

C-index, Harrell's concordance index.

Care level 1, care level 2, and the composite outcome (care level 2 or death) were defined only in KAWP; ADL disability outcomes were defined only in TOOTH.

In TOOTH, comparable associations were observed for all-cause mortality (HR 0.74, 0.60–0.91) and the composite of ADL disability or death (HR 0.78, 0.67–0.91), with adjusted C-indices of 0.62 and 0.61, respectively. For ADL disability alone, the association was in the same direction but not statistically significant (HR 0.84, 0.66–1.07).

### 3.5 Competing-risk analysis

Table S4 presents the results of Fine-Gray competing-risk models in which death was treated as a competing event. In KAWP, the association between IC and disability was substantially attenuated, with significance no longer maintained (care level 1: adjusted subdistribution HR [SHR] 1.01, 0.92–1.12; care level 2: SHR 0.97, 0.81–1.17). A similarly null association was observed in TOOTH for ADL disability (SHR 0.97, 0.73–1.30). Thus, the association between IC and disability was no longer distinguishable from the null when death was treated as a competing event rather than as censoring.

### 3.6 Model calibration and the incremental contribution of the cardio-renal measure

Model performance, as assessed by the scaled integrated Brier score, was limited across outcomes. In KAWP, scaled integrated Brier scores for the five-domain IC model ranged from 0.030 to 0.050, whereas corresponding values in TOOTH were 0.021 for all-cause mortality and 0.018 for the composite outcome (Table S5). The incremental prognostic contribution of the cardio-renal measure was then evaluated using two complementary approaches. First, recomputing a six-domain composite that added the cardio-renal measure as an equally weighted sixth component to create an extended composite resulted in only marginal improvements in discrimination, with an increase in the C-index of ≤0.012 across all outcomes. For all-cause mortality in KAWP the C-index increased from 0.666 to 0.675 (ΔC-index 0.010, 0.004–0.015; Table S3). Second, when the standardized cardio-renal measure was entered alongside the intact five-domain IC score in nested models, it demonstrated a consistent incremental association with outcomes, most prominently with mortality: in KAWP, each SD higher cardio-renal score was associated with a lower risk of all-cause mortality (HR 0.71, 0.63–0.79; likelihood ratio test *p* < 0.001). A comparable association was observed in TOOTH (HR 0.72, 0.59–0.88; *p* = 0.002; Table S6). The gain in discrimination in the nested model was small but, for the composite outcome and all-cause mortality in KAWP, statistically significant (ΔC-index 0.023, 0.008–0.039, and 0.027, 0.010–0.046, respectively). Notably, the improvement in discrimination for mortality in KAWP was larger when the cardio-renal measure was modeled as a separate component alongside the five-domain IC score (ΔC-index 0.027) than when it was incorporated into the equally weighted six-domain composite (ΔC-index 0.010). Estimates for the five-domain IC score were modestly attenuated after the inclusion of the cardio-renal measure, most noticeably for all-cause mortality in TOOTH (HR 0.74 to 0.80).

## 4 Discussion

This study examined the associations of IC with disability and all-cause mortality among community-dwelling octogenarians across two independent Japanese cohorts. Three principal findings emerged. First, higher IC was consistently associated with lower risks of disability and all-cause mortality, and these associations changed little after adjustment for potential confounders. Second, the direction and magnitude of these associations were broadly concordant across two independently analyzed cohorts that differ in setting, calendar period, and disability ascertainment. Third, and most notably, the association between IC and disability was no longer evident after accounting for death as a competing event. These findings suggest that IC in this age group is better understood as a marker of shared vulnerability to both disability and death than as a predictor of disability independent of mortality. The cardio-renal measure added a consistent but small amount of prognostic information beyond the five domains, chiefly for mortality.

Higher IC was consistently associated with lower risks of disability and mortality among Japanese octogenarians, aligning with previous longitudinal studies demonstrating that lower IC is associated with adverse health outcomes in older populations [5,[37], [38], [39]]. Consistent with previous studies, locomotor and cognitive domains showed the strongest associations with disability and mortality, whereas sensory or psychological domains were weaker or less consistent [13,15,40]. Because the domains were not mutually adjusted, these comparisons describe each domain considered on its own rather than its independent contribution. Notably, our study extends previous work by focusing particularly on the oldest old, whereas most earlier studies comprised adults aged 60 or 65 years and older [1,14,38]. Our findings suggest that the prognostic contribution of IC domains may become more heterogeneous with advancing age, highlighting the potential need for additional indicators of physiological reserve to complement conventional IC domains in assessing health risks in the oldest old.

A key strength of this study was the consistency of the IC–outcome associations across two independently analyzed cohorts of community-dwelling octogenarians recruited in different settings and time periods. Because disability was ascertained differently in the two studies, this cross-cohort consistency does not constitute formal replication or external validation. Nevertheless, it suggests that the observed associations are not an artifact of a single outcome definition and that the IC framework captures fundamental aspects of vulnerability that remain prognostically relevant in very advanced age, in which competing risks and multimorbidity are pervasive.

An important and novel observation was the marked attenuation of the IC-disability association in competing-risk analyses, which is consistent with the wider literature on older adults. IC is associated with both later disability or dependence and mortality, and meta-analyses and longitudinal cohorts have found that greater IC is associated with lower risks of basic and instrumental activities of everyday living impairment, dependence, nursing home admission, and death [5,6,38]. In the oldest old, this matters because many individuals with low IC are also at high risk of death before disability is noticed or formally confirmed. Studies of older adults have reported that when death is common during follow-up, standard Kaplan–Meier or Cox approaches can overestimate the incidence of non-fatal outcomes if death is treated as simple censoring rather than as a competing event [41,42]. This concern is particularly relevant in cohorts with long follow-up and high cumulative mortality, in which the bias introduced by competing death becomes most substantive [43,44]. Methodological work in older populations has shown that the cumulative incidence function and subdistribution hazard models are more appropriate for prediction questions in the presence of competing events, whereas cause-specific models answer a different question regarding the rate of disability among those still alive and event-free [42,45]. Our findings are broadly consistent with previous literature: the near-null subdistribution hazards for disability, contrasted with the robust cause-specific associations and the strong association between IC and mortality, indicate that these two estimands answer different questions at these ages. Among individuals who remained alive and disability-free, higher IC was associated with a lower rate of disability; on the cumulative incidence scale, however, disability risk differed little by IC, because those with lower IC were also more likely to die before disability occurred. IC in advanced age might therefore be best understood as a marker of overall vulnerability capturing the shared risk of disability and death rather than of disability independent of mortality. These findings underscore the value of reporting both cause-specific and subdistribution estimates when predicting non-fatal outcomes in populations with substantial competing mortality.

We also explored whether a cardio-renal measure based on NT-proBNP and cystatin C added prognostic information beyond the five conventional domains. Rather than recomputing a six-domain composite—which would alter the weighting of the five constituent domains and conflate the addition of new information with a change in the scaling of existing information—we entered the cardio-renal measure alongside the five-domain score in nested models. This yielded a consistent contribution, reaching statistical significance for all outcomes in KAWP and for two of three outcomes in TOOTH, with the strongest association observed for mortality, for which its magnitude was comparable to that of the IC score itself. Nevertheless, improvements in discrimination were small, with a maximum increase of 0.027 in the C-index, and the resulting models remained unsuitable for individual-level prediction. This pattern is consistent with prior work extending the WHO IC framework by incorporating comorbidity or biomarker information, which has generally reported only modest incremental gains [8,9,[11], [12], [13]]. We therefore interpret the cardio-renal measure as providing complementary prognostic information beyond the five conventional IC domains, particularly for survival, rather than evidence that biomarkers should be incorporated into the IC framework as currently conceived. Whether biomarker-based physiological indicators are better used alongside, rather than within, the IC framework warrants further investigation.

These findings have several implications for practice and policy. They support the continued use of IC within the WHO Integrated Care for Older People framework as a broad screening construct for identifying vulnerability at the population level among adults aged 85 years and older and are consistent with the emphasis that Integrated Care for Older People places on locomotor and cognitive function. However, our findings do not establish IC as a stand-alone prediction tool: discrimination was modest in both cohorts, and the present data do not support the use of a specific clinical cut-off for individual risk stratification, nor do they demonstrate that interventions targeting particular IC domains would reduce disability or mortality, which would require evidence from intervention trials. Finally, the competing-risk findings have practical implications for programs aimed at preventing disability at very advanced ages. Because death is the more frequent competing outcome, the achievable benefit of disability prevention is bounded by survival. Accordingly, interventions in this population should be evaluated with respect to both disability and mortality rather than disability alone.

This study has several strengths. First, we analyzed data from two well-characterized cohort studies of adults aged 85–89 years, allowing for a primary analysis and an assessment of consistency in an independently analyzed cohort. Second, we incorporated multidimensional assessments of IC spanning objective physical performance, cognitive function, psychological wellbeing, sensory function, and physiological biomarkers. Third, we evaluated clinically relevant outcomes, including disability and mortality, which are key indicators of healthy aging in very old populations. Fourth, we applied a harmonized set of covariates across both cohorts and used competing-risk models and calibration measures in addition to conventional discrimination, providing a more comprehensive and consistent assessment of prognostic performance. The observed associations were robust to adjustment for potential confounders and remained consistent when the same covariate set was applied to individual IC domains. Several limitations should be considered. First, some domains were assessed using self-reported measures, which may introduce measurement error. Second, disability was defined differently in the two cohorts—using administrative long-term care certification in KAWP and Barthel Index-based ADL dependency in TOOTH—which limits direct comparability. Nevertheless, the concordant findings across these distinct definitions provide support for the robustness of the observed associations. Third, ADL in TOOTH was assessed only at discrete 3- and 6-year visits; consequently, the timing of ADL disability onset was interval-censored, and findings based on ADL disability alone should therefore be interpreted with caution. Fourth, participants excluded because of missing data in TOOTH were older and had lower functions, suggesting that the analytic sample may have been modestly healthier than the underlying cohort. Fifth, the psychological domain was indexed by the WHO-5, which captures subjective well-being rather than depressive symptomatology; the weaker associations observed for this domain may therefore partly reflect the instrument used. Sixth, the incremental improvement from adding the cardio-renal measure was small and should not be overinterpreted. Finally, the study population consisted of very old Japanese adults, which may limit generalizability to younger populations or other ethnic groups.

Several directions follow from this work. Harmonized measurement of IC and disability across cohorts of the oldest old would allow associations to be compared directly rather than assessed only for consistency. Repeated assessment of IC would enable trajectories, rather than a single baseline measure, to be examined in relation to outcomes and may be particularly informative at ages when capacity changes rapidly. Formal external validation in populations outside Japan is needed before any predictive application can be considered. Analyses of non-fatal outcomes in this age group should report both cause-specific and subdistribution estimates, as these approaches address different questions and, as shown here, may yield different conclusions. Finally, whether biomarker-based indicators of physiological reserve are better used alongside the IC framework or incorporated into it warrants direct evaluation.

## 5 Conclusions

In community-dwelling octogenarians, higher IC was consistently associated with lower risks of disability and mortality, with broadly consistent associations across two independently analyzed Japanese cohorts. When the individual domains were examined separately, locomotor and cognitive capacity demonstrated the strongest associations with both outcomes. Notably, the association between IC and disability was no longer evident when death was treated as a competing risk, underscoring that IC at these ages captures shared vulnerability to disability and death rather than disability independent of mortality. Model discrimination remained limited across analyses, providing little support for the use of IC as a tool for individual-level risk stratification. An exploratory cardio-renal measure added a small but consistent increment in prognostic information beyond the five conventional IC domains, particularly for mortality.

## Ethics approval and consent to participate

This study was conducted in accordance with the Declaration of Helsinki. Ethical approval was obtained from the Ethics Committee of Keio University School of Medicine for the KAWP study (approval no. 20160297) and the TOOTH study (approval no. 20070047). Written informed consent was obtained from all participants prior to enrollment.

## Availability of data and materials

The dataset analyzed during the present study is not publicly available, as it includes sensitive information from a vulnerable population. However, they are available from the last author on reasonable request and with approval from the Research Ethics Committee of Keio University School of Medicine. To request access to the data, please contact Dr. Yasumichi Arai (yasumich@keio.jp).

## Funding

This work was supported by JSPS KAKENHI (Grant Number 24K02711), JST Research Complex Program (JP15667051), and grants from the Kanagawa Institute of Industrial Science and Technology (KISTEC). The funders had no role in the study design, data collection, analysis, interpretation, or preparation of the manuscript.

## Declaration of Generative AI and AI-assisted technologies in the writing process

The analysis plan and all statistical decisions, including model specification, covariate selection and the handling of competing risks, were determined by the authors before analysis. Generative AI (Claude Opus 5, Anthropic) was used solely to assist with developing and debugging the R code used for the statistical analyses (Fine-Gray competing-risk models and model calibration). The first author reviewed the code line by line against the analysis plan, and a co-author independently verified the reported estimates against the analysis output. All authors take full responsibility for the analyses.

## Clinical trial number

University Hospital Medical Information Network Clinical Trials Registry under UMIN000026053 for KAWP and UMIN000001842 for TOOTH.

## CRediT authorship contribution statement

**Yusuke Osawa:** Writing – original draft, Visualization, Methodology, Investigation, Funding acquisition, Formal analysis, Conceptualization. **Takashi Sasaki:** Writing – review & editing, Investigation, Data curation. **Yukiko Abe:** Writing – review & editing, Investigation, Data curation. **Yasumichi Arai:** Writing – review & editing, Supervision, Project administration, Methodology, Investigation, Funding acquisition, Conceptualization.

## Declaration of competing interest

The authors declare that they have no known competing financial interests or personal relationships that could have appeared to influence the work reported in this paper.
