# Supplementary material for: Intrinsic capacity and risks of disability and mortality in octogenarians: a prospective analysis of two independent Japanese cohorts
Source: J Frailty Aging. 2026 Nov 5;15(6):100230. doi: 10.1016/j.tjfa.2026.100230 (PMC13757523; doi:10.1016/j.tjfa.2026.100230)
Supplement: Supplementary file 1 [file mmc1.docx]

**Supplementary materials**

**Table S1. Baseline characteristics of participants included in and excluded from the analytic samples, by cohort**

|  | **KAWP** | | |  | **TOOTH** | | |
| --- | --- | --- | --- | --- | --- | --- | --- |
|  | **Included** | **Excluded** | **p-value** |  | **Included** | **Excluded** | **p-value** |
| **Variable** | (n = 942) | (n = 84) |  |  | (n = 393) | (n = 97) |  |
| Age, years, mean (SD) | 86.6 (1.4) | 86.5 (1.3) | 0.839 |  | 87.3 (1.3) | 87.6 (1.3) | 0.041 |
| Female, n (%) | 480 (51.0) | 33 (39.3) | 0.053 |  | 218 (55.5) | 57 (58.8) | 0.638 |
| BMI, kg/m^2^, mean (SD) | 23.3 (3.1) | 22.8 (3.2) | 0.190 |  | 21.6 (3.2) | 21.2 (3.3) | 0.295 |
| Education (HS+), n (%) | 382 (40.6) | 24 (28.6) | 0.042 |  | 151 (38.4) | 26 (26.8) | 0.044 |
| Ever smoker, n (%) | 394 (41.8) | 33 (41.2) | 1.000 |  | 162 (41.2) | 34 (40.5) | 0.997 |
| TUG, sec, mean (SD) | 11.6 (2.9) | 11.6 (3.5) | 0.419 |  | 14.9 (8.7) | 16.3 (6.5) | 0.117 |
| MMSE, points, mean (SD) | 26.1 (2.8) | 25.5 (3.1) | 0.140 |  | 26.8 (3.2) | 24.5 (5.7) | 0.003 |
| WHO-5, points, mean (SD) | 18.3 (5.1) | 18.6 (5.4) | 0.633 |  | 18.7 (5.2) | 15.8 (5.4) | <0.001 |
| Grip strength, kg, mean (SD) | 23.5 (6.3) | 22.7 (6.3) | 0.302 |  | 20.6 (5.6) | 19.0 (6.6) | 0.013 |
| Vision score, median [IQR] | 3 [3−3] | 3 [2−3] | 0.597 |  | 4 [4−4] | 4 [3−4] | <0.001 |
| Hearing score, median [IQR] | 3 [3−4] | 3 [3−4] | 0.667 |  | 3 [2−3] | 3 [2−3] | 0.283 |
| NT-proBNP, pg/mL, median [IQR] | 144.0  [87.0−258.8] | 168.0  [106.5−290.5] | 0.166 |  | 191.0  [113.5−376.5] | 180.0  [112.5−420.2] | 0.927 |
| Cystatin C, mg/L, median [IQR] | 1.09  [0.95−1.26] | 1.17 [0.95−1.28] | 0.203 |  | 1.17  [1.02−1.38] | 1.13  [1.04−1.27] | 0.540 |

BMI, body mass index; MMSE, Mini-Mental State Examination; TUG, Timed Up and Go test; WHO-5, WHO Well-being Index.

KAWP: baseline participants aged 85-89 years (n = 1,026). TOOTH: participants aged 85-89 years (n = 490).

**Table S2**. **Domain measures in intrinsic capacity in both cohorts**

| **Domain** | **Instrument** | **Scoring range** | **Direction** |
| --- | --- | --- | --- |
| Locomotor | Timed-up and Go test | seconds (continuous) | lower TUG = better |
| Cognitive | Mini-Mental State Examination (MMSE) | 0−30 points | higher = better |
| Psychological | WHO-5 Well-being Index | 0−25 points | higher = better |
| Sensory (vision) | Self-rated vision (single item) | KAWP: 5 levels; TOOTH: 4 levels | reverse-coded so higher = better |
| Sensory (hearing) | Self-rated hearing (single item) | 4 levels (both cohorts) | reverse-coded so higher = better |
| Vitality | Handgrip strength (kg) | kg (continuous) | higher = better |
| Each domain measure was standardized within cohort to 0-100 (higher = better) and the five domain scores were averaged into the composite IC score. | | | |
| The cardio-renal measure (exploratory) was derived from log-transformed NT-proBNP and cystatin C, standardized, summed, and reversed so higher = better reserve. | | | |

**Table S3. Discrimination of the five-domain and recomputed six-domain intrinsic capacity scores for disability and all-cause mortality**

| **Cohort** | **Outcome** | **C-index 5-domain** | **C-index 6-domain** | **Delta C-index (95% CI)** |  |
| --- | --- | --- | --- | --- | --- |
| KAWP | Care level 1 | 0.610 | 0.615 | 0.004 (-0.001−0.009) | |
|  | Care level 2 | 0.611 | 0.619 | 0.008 (0.000−0.016) | |
|  | Composite | 0.615 | 0.626 | 0.011 (0.005−0.018) | |
|  | All-cause mortality | 0.666 | 0.675 | 0.010 (0.004−0.015) | |
| TOOTH | All-cause mortality | 0.609 | 0.621 | 0.012 (0.000−0.019) | |
|  | ADL disability | 0.682 | 0.687 | 0.005 (-0.003−0.015) | |
|  | ADL disability or death | 0.599 | 0.607 | 0.007 (-0.001−0.015) | |
| Comparison of the five-domain IC score with a recomputed six-domain composite that adds the cardio-renal measure as an equally weighted sixth component. All models adjusted for age, sex, education, smoking, comorbidity, and BMI. | | | | | |
| Delta C-index: change in Harrell's C-index from the five- to six-domain composite, with bootstrap 95% CI (1000 resamples). Recomputing the composite dilutes the weight of the five original domains, so gains are small. | | | | | |
| In KAWP, both scores were estimated in all 942 participants. In TOOTH, the six-domain composite cannot be computed when either biomarker is missing, so both scores were estimated in the 360 participants with complete NT-proBNP and cystatin C data; the five-domain C-indices shown here therefore differ slightly from those in Table 2, which are based on all 393 participants. | | | | | |
| Delta C-index is the bootstrap point estimate and may differ by up to 0.001 from the difference between the two rounded C-indices shown. | | | | | |

**Table S4. Fine–Gray subdistribution hazard models for incident disability with death as a competing event**

|  |  |  |  | **5-domain intrinsic capacity** | | **6-domain intrinsic capacity** | |
| --- | --- | --- | --- | --- | --- | --- | --- |
|  |  |  |  | **Base model** | **Fully adjusted model** | **Base model** | **Fully adjusted model** |
| **Cohort** | **Outcome** | **N** | **Events** | **SHR (95%CI)** | **SHR (95%CI)** | **SHR (95%CI)** | **SHR (95%CI)** |
| KAWP | Care 1 | 942 | 656 | 1.02 (0.93−1.13) | 1.01 (0.92−1.12) | 1.02 (0.91−1.13) | 1.00 (0.90−1.12) |
|  | Care 2 | 942 | 276 | 0.97 (0.81−1.16) | 0.97 (0.81−1.17) | 0.95 (0.77−1.16) | 0.95 (0.77−1.16) |
| TOOTH | ADL disability  (death as competing) | 393 | 88 | 0.99 (0.75−1.31) | 0.97 (0.73−1.30) | 0.94 (0.70−1.27) | 0.94 (0.70−1.26) |
| Subdistribution hazard ratios (SHR) and 95% confidence intervals (CI) from Fine-Gray competing-risk models, expressed per 10-point difference in the IC score, with death treated as the competing event. | | | | | | | |
| Base model: adjusted for age and sex. Fully adjusted model: additionally adjusted for education, smoking status, Charlson Comorbidity Index, and body mass index. | | | | | | | |
| In KAWP, competing-risk models were fitted for care level 1 and care level 2. In TOOTH, the model was fitted for ADL disability, with death as the competing event. | | | | | | | |
| N and Events refer to the five-domain models. In TOOTH, the six-domain models were fitted in the 360 participants with complete NT-proBNP and cystatin C data (82 ADL disability events). | | | | | | | |

**Table S5. Calibration of the five-domain and six-domain intrinsic capacity models (integrated Brier score)**

| **Cohort** | **Outcome** | **Score** | **IBS (null)** | **IBS (Cox)** | **Scaled IBS** |
| --- | --- | --- | --- | --- | --- |
| KAWP | Care level 1 | 5-domain | 0.2082 | 0.1979 | 0.0496 |
|  | Care level 2 |  | 0.0992 | 0.0963 | 0.0298 |
|  | Composite |  | 0.1340 | 0.1286 | 0.0402 |
|  | All-cause mortality |  | 0.1066 | 0.1021 | 0.0428 |
|  | Care level 1 | 6-domain | 0.2082 | 0.1968 | 0.0549 |
|  | Care level 2 |  | 0.0992 | 0.0957 | 0.0351 |
|  | Composite |  | 0.1340 | 0.1272 | 0.0508 |
|  | All-cause mortality |  | 0.1066 | 0.1014 | 0.0487 |
| TOOTH | All-cause mortality | 5-domain | 0.1187 | 0.1163 | 0.0207 |
|  | ADL or death |  | 0.1187 | 0.1165 | 0.0184 |
|  | All-cause mortality | 6-domain | 0.1186 | 0.1148 | 0.0322 |
|  | ADL or death |  | 0.1186 | 0.1150 | 0.0303 |
| IBS, integrated Brier score, a measure of model calibration (lower values indicate better calibration); IBS (Cox) corresponds to the fully adjusted Cox model. | | | | | |
| Scaled IBS = 1 - (IBS Cox / IBS null), analogous to an R-squared measure of predictive accuracy. Higher values indicate greater improvement over the null model. | | | | | |
| 5-domain and 6-domain refer to the five-domain and six-domain intrinsic capacity scores, respectively. | | | | | |

**Table S6. Nested models entering the cardio-renal measure alongside the intact five-domain intrinsic capacity score**

|  |  |  |  | **Base model** | **Extended model** | |  |  |  |  |  |
| --- | --- | --- | --- | --- | --- | --- | --- | --- | --- | --- | --- |
| **Cohort** | **Outcome** | **N** | **Events** | **HR (95%CI)** | **HR (95%CI)** | **HR (95%CI) cardio-renal measure** | **C-index** | **C-index** | **ΔC-index** | **LRT** |  |
|  |  |  |  |  | **Five-domain IC** |  | **Base** | **EX** |  | **p-value** |  |
| KAWP | Care level 1 | 942 | 656 | 0.72 | 0.73 | 0.91 | 0.610 | 0.615 | 0.005  (-0.000 − 0.014) | 0.023 |  |
|  |  |  |  | (0.66−0.79) | (0.67−0.80) | (0.84−0.99) |  |  |  |  |  |
|  | Care level 2 |  | 276 | 0.67 | 0.69 | 0.84 | 0.611 | 0.622 | 0.011  (0.000 − 0.030) | 0.005 |  |
|  |  |  |  | (0.58−0.77) | (0.60−0.79) | (0.74−0.94) |  |  |  |  |  |
|  | Composite |  | 425 | 0.71 | 0.74 | 0.77 | 0.615 | 0.638 | 0.023  (0.008 − 0.039) | <0.001 |  |
|  |  |  |  | (0.64−0.79) | (0.67−0.83) | (0.70−0.84) |  |  |  |  |  |
|  | All-cause mortality |  | 257 | 0.78 | 0.83 | 0.71 | 0.666 | 0.693 | 0.027  (0.010 − 0.046) | <0.001 |  |
|  |  |  |  | (0.68−0.90) | (0.72−0.96) | (0.63−0.79) |  |  |  |  |  |
| TOOTH | All-cause mortality | 360 | 107 | 0.74 | 0.80 | 0.72 | 0.609 | 0.628 | 0.019  (-0.004 to 0.053) | 0.002 |  |
|  |  |  |  | (0.59−0.91) | (0.63−1.00) | (0.59−0.88) |  |  |  |  |  |
|  | ADL disability |  | 82 | 0.83 | 0.88 | 0.81 | 0.682 | 0.698 | 0.015  (-0.003 − 0.063) | 0.100 |  |
|  |  |  |  | (0.64−1.06) | (0.67−1.15) | (0.63−1.03) |  |  |  |  |  |
|  | ADL disability |  | 189 | 0.77 | 0.83 | 0.76 | 0.599 | 0.614 | 0.014  (-0.006 − 0.050) | 0.001 |  |
|  | or death |  |  | (0.65−0.91) | (0.70−0.99) | (0.65−0.89) |  |  |  |  |  |
| The base model contains the five-domain IC score (per 10-point higher) plus age, sex, education, smoking, Charlson Comorbidity Index and body mass index. | | | | | | | | | | | |
| The extended model adds the standardized cardio-renal measure (per 1 SD higher, derived from NT-proBNP and cystatin C) to the same model, so that its incremental contribution is estimated without altering the weighting of the five constituent domains. | | | | | | | | | | | |
| LRT p-value: likelihood ratio test comparing the two nested models. ΔC-index: difference in Harrell's concordance index. | | | | | | | | | | | |
| In TOOTH, models were fitted in the 360 participants with complete NT-proBNP and cystatin C data; the five-domain estimates in Table 2 are based on all 393 participants. | | | | | | | | | | | |
|  |  |  |  |  |  |  |  |  |  |  |  |

**Supplementary Figure S1.** Kaplan–Meier curves of the five-domain intrinsic capacity score, categorized by tertile according to all-cause mortality (left panel) and the composite outcome of activities of daily living disability or death (right panel) in the Tokyo Oldest-Old Survey on Total Health
Abbreviations: IC, intrinsic capacity
